# Herpes zoster vaccination and the risk of dementia: A systematic review and meta‐analysis

**DOI:** 10.1002/brb3.3415

**Published:** 2024-02-05

**Authors:** Sangam Shah, Krishna Dahal, Sangharsha Thapa, Prativa Subedi, Basanta Sharma Paudel, Swati Chand, Amr Salem, Markus Lammle, Ranjit Sah, Martin Krsak

**Affiliations:** ^1^ Institute of Medicine Tribhuvan University Maharajgunj Nepal; ^2^ Westchester Medical Center Valhalla New York USA; ^3^ Kist Medical College and Teaching Hospital Imadole, Lalitpur Nepal; ^4^ SUNY Upstate Medical University Syracuse New York USA; ^5^ Department of Microbiology, Tribhuvan University Teaching Hospital Institute of Medicine Kathmandu Nepal; ^6^ Department of Microbiology, Dr. D. Y. Patil Medical College, Hospital and Research Centre Dr. D. Y. Patil Vidyapeeth Pune Maharashtra India; ^7^ Datta Meghe Institute of Higher Education and Research Jawaharlal Nehru Medical College Wardha India; ^8^ Division of Infectious Diseases University of Colorado Anschutz Medical Campus Aurora Colorado USA

**Keywords:** dementia, herpes zoster, shingles, vaccination

## Abstract

**Introduction:**

Previous studies have reported a decreased risk of dementia with herpes zoster vaccination. Given this background, this systematic review and meta‐analysis aimed to investigate the association between herpes zoster vaccination and the risk of dementia.

**Methods:**

We searched five databases until November 2023 for case–control, cross‐sectional, or cohort studies investigating the association of herpes zoster vaccination and dementia. Odds ratios and 95% confidence intervals (95% CIs) were pooled in the meta‐analysis. Meta‐regression, subgroup, and sensitivity analysis were also conducted.

**Results:**

We evaluated a total of five studies (one cross‐sectional, one case–control, and four cohort studies) that included a total number of 103,615 patients who were vaccinated with herpes zoster vaccine. All the studies were of high quality, ranging from 7 to 9. Due to the high heterogeneity (*I* ^2^ = 100%, *p* < .00001) observed in our study, a random effect model was used for the analysis. The pooled odds ratio was 0.84 (95% CI: 0.50, 1.43), *p* (overall effect) = .53), indicating that herpes zoster vaccination reduces the risk of dementia.

**Conclusion:**

Herpes zoster vaccination is associated with a reduction of the risk of dementia. More epidemiological studies are needed to confirm the association.

## Graphical abstract

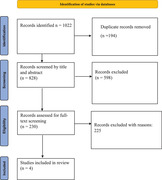

## 1 INTRODUCTION

Herpes zoster, commonly known as shingles, is a viral infection caused by the reactivation of the varicella‐zoster virus (VZV), the same virus responsible for chickenpox (varicella) (Centers for Disease Control and Prevention, 2023a). After an individual recovers from chickenpox, the VZV remains dormant in the dorsal nerve root ganglia which, when reactivated, causes herpes zoster and is characterized by grouped vesicular rashes (Centers for Disease Control and Prevention, 2023).

Vaccines are available for herpes zoster and post‐herpetic neuralgia, a live attenuated VZ virus (VZV) vaccine ZVL (zoster vaccine live); ZOSTAVAX (Merck) and VZV glycoprotein E (adjuvanted, gE) subunit vaccine recombinant ZV (RZV); ShINGRIX (GlaxoSmithkline). A number of studies have shown that both ZVL and RZV are safe and efficacious. RZV is safe among immunocompromised people due to its non‐replicating nature. For the purpose of preventing HZ and its consequences, including post herpetic neuralgia (PHN), the Advisory Committee on Immunization Practices (ACIP) advised routine ZVL (ZOSTAVAX; Merck) treatment to people ≥60 years of age in 2006. However, the ACIP recommended RZV for immunocompetent people ≥50 years of age in 2018 because of its greater efficacy (Dooling et al., 2018). The varicella zoster vaccine contains a minimum of 19,400 PFU of the Oka/Merck strain of the varicella virus (Centers for Disease Control and Prevention, 2023b). The minimum potency of the zoster vaccine is at least 14 times the potency of the varicella vaccine (Varivax), which contains a minimum of 1350 PFU, and is similar in potency to the varicella zoster virus content of measles–mumps–rubella–varicella vaccine (ProQuad) (Centers for Disease Control and Prevention, 2023b). In the elderly population, widely available zoster vaccines have been a blessing for their health (Harbecke et al., 2023).

The relationship between HZ and dementia is still controversial. The likely pathophysiology of dementia could be due to the process called neuroinflammation, involving the formation of misfolded oligomers, amyloid plaque accumulation, and hyperphosphorylated Tau protein containing neurofibrillary tangles (Johannesdottir Schmidt et al., 2022). This may result from direct neural damage or cerebral vasculopathy. In some hypotheses, astrocytes may be infected directly, leading to the generation of intracellular amyloid and the aggregation of amyloid fibrils in the extracellular environment (Johannesdottir Schmidt et al., 2022). Another possibility suggests that during the involvement of cranial nerves due to HZ, cerebral vasculopathy and stroke may occur, leading to neural damage (Johannesdottir Schmidt et al., 2022).

Several studies show that HZ is associated with a slight increase in the risk of dementia when antivirals are administered, but it also appears to lower the risk of dementia development in individuals with HZ (Chen et al., 2018). One study by Bae et al. (2021) demonstrated a positive relationship between HZ and dementia, as well as the effect of antivirals in reducing the risk of dementia in HZ patients. Another study by Tsai et al. (2017) found that patients with herpes zoster ophthalmicus are linked with a higher risk of dementia. However, the study by Choi et al. (2021) did not find an increased risk of dementia due to HZ. Nevertheless, these postulations lack sufficient evidence to fully support them. Therefore, this systematic review and meta‐analysis aimed to investigate the association between herpes zoster vaccination and the risk of dementia

## 2 METHODOLOGY

The Preferred Reporting Items for Systematic Reviews and Meta‐Analyses and Cochrane Handbook of systematic review standards were followed for conducting this meta‐analysis (Green, 2019; Liberati et al., 2009).

### 2.1 Search strategy

Databases from their inception to November 2023 were thoroughly searched, including PubMed, EMBASE, Cochrane Library, clinicaltrials.gov, and Web of Science. No language constraints were applied, and both keywords and medical topic headings (MeSH) were used in the search approach. The study's search criteria were “herpes zoster” OR “shingles” OR “HZV” OR “varicella zoster virus” OR “vaccine*” AND “Dementia.” Additionally, we manually searched reference lists and evaluations of related research that were included in order to find other papers that were pertinent.

### 2.2 Eligibility criteria

According to the following eligibility requirements, studies were included: (1) cohort, case–control, or cross‐sectional study designs and (2) dementia reports as the result. When multiple data from a single study were presented, we took the data from the study with the longest follow‐up or considered the most participants.

### 2.3 Exclusion criteria

The following types of studies were not included: (1) conference abstracts or research protocols, (2) publications that were published twice, (3) studies with insufficient or no results, (4) articles not mentioning dementia, and (5) non‐English language articles.

### 2.4 Research selection

Using eligibility and exclusion criteria, two reviewers independently assessed the literature included in this investigation. First, duplicate publications were eliminated, as well as those deemed irrelevant to this study based on titles and abstracts. Afterward, potential articles were found, downloaded, and thoroughly studied to make sure the data could be properly integrated. In cases where there was debate about whether or not to include an article, the two researchers talked with a third reviewer to come to a decision.

### 2.5 Data extraction

The first author, year of publication, country, study type, study design, sample size, participant age, participant gender, and dementia diagnosis were all retrieved and summarized independently by the two authors from each of the included studies. Another author verified the extracted data.

### 2.6 Risk of bias assessment

The Newcastle–Ottawa Scale (NOS) for nonrandomized studies was employed to rate the research quality. The NOS is a method of rating studies in which points are assigned depending on the degree to which a researcher's objectives are met in the three distinct domains of selection, comparability, and outcome (Lo et al., 2014). Two researchers carried out the scoring. The final step was to calculate the overall score, with 7–9 being regarded as a good‐quality study with a low chance of bias, 4–6 as a high risk of bias, and 0–3 as an extremely high risk of bias. Any disagreements that arose during the risk of bias assessment were resolved by the supervisor.

### 2.7 Statistical analysis

We took the adjusted odds ratio (OR) and 95% confidence interval (CI) from each trial to analyze the relationship between the herpes vaccine and the risk of dementia. Statistical analysis was performed on the retrieved adjusted OR and 95% CI using Stata v. 16.0 (Stata Corp.). The chi‐square test and *I* ^2^ were used to determine how heterogeneous the included studies were. Statistical heterogeneity was regarded as nonsignificant when *p* was >0.05. The random‐effects model was used if I2 > 50%, that indicated considerable heterogeneity and fixed‐effects model was employed when I2 was >50%, or P 0.1 indicated considerable heterogeneity. Through subgroup analysis and sensitivity analysis, the robustness of the results with regard to the overall effect of statistics and sources of heterogeneity was confirmed. The visual inspection of funnel plots was utilized to validate publication bias, and the Egger regression test was applied to quantitatively detect publication bias for asymmetry.

## 3 RESULTS

### 3.1 Study selection

**FIGURE 1:**
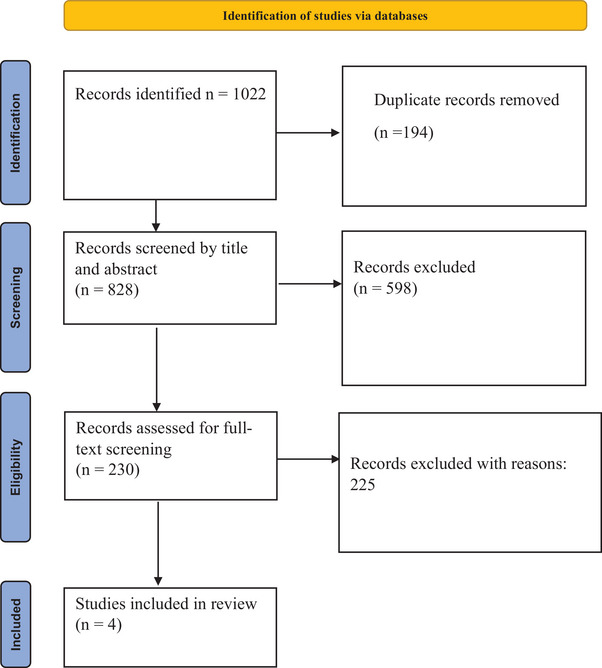
Preferred Reporting Items for Systematic Reviews and Meta‐Analyses (PRISMA) flowchart of the included studies.

There were 1022 studies found in the literature search. Following the elimination of duplicate articles, the remaining articles were subjected to title and abstract screening, full‐text screening, and finally, inclusion of five studies that met the inclusion criteria for both qualitative and quantitative syntheses. Figure 1 shows the full list of studies selections.

### 3.2 Study characteristics

The characteristics of the included studies (*n* = 4) are summarized in Table 1. All studies were retrospective in nature and were published in the years 2021 and 2023. The studies included are cohort study (*n* = 2), case–control study (*n* = 1), and cross‐sectional study (*n* = 1). The studies were conducted in United States (*n* = 2) (Lehrer & Rheinstein, 2021; Scherrer et al., 2021) or in the United Kingdom (*n* = 2) (Lophatananon et al., 2023, 2021). A total number of 941,991 patients were vaccinated with herpes zoster vaccine.

**TABLE 1:** Descriptive characteristics of the included studies.

Serial No.	Author and year of study	Study country	Study design	Population vaccinated (number)	Population non vaccinated	Age of vaccinated population (years)	Gender distribution of vaccinated population (male: female)	Vaccine(s) employed	Definition for dementia
1.	Lehrer et al. 2021 (Lehrer & Rheinstein, 2021)	United States	Cross‐sectional, interviewed	99	176	70 ± 7	33:37	Zostavax (Merek)/ Shingrix (GDK)	Social activities hampered by disorientation or memory loss
2.	Lophatananon et al. 2021 (Lophatananon et al., 2021)	United Kingdom	Case–control Study	35,116	193,020	≥70	45.59:54.41	Zostavax	ICD 9 and 10 codes
3.	Wiemken et al. 2021 (Wiemken et al., 2021)	United States	Retrospective two‐cohort study	27,419 in Veterans Health Affairs (VHA) cohort, 24,612 in market scan cohort	108,597	**≥**65 Years	95.9:4.1 for VHA cohort, 37.3:62.7 for market scan cohort	Proquad/ Varivax/Zostavax/Shingrix	ICD 9 and 10 codes
4.	Lophatananon et al. 2023 (Lophatananon et al., 2023)	United Kingdom	Retrospective cohort study	854,745	8,490,813	≥70	47.7:52.3	–	ICD 10 codes

### 3.3 Bias assessment

**FIGURE 2:**
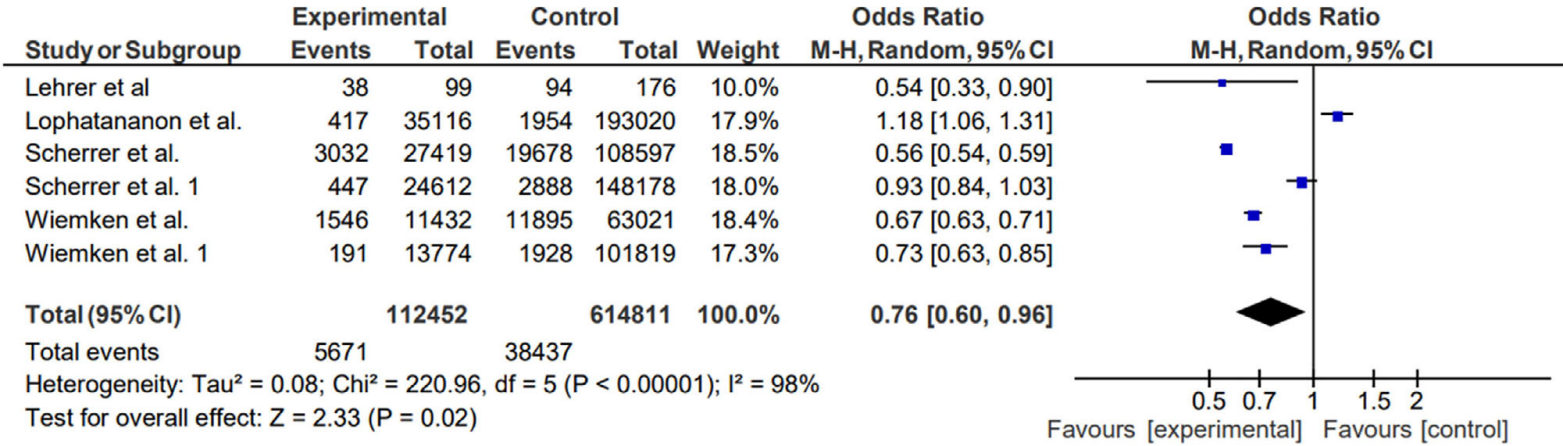
Forest plot showing risk of dementia after herpes zoster vaccination.

Table 2 displays the findings of the studies' assessment of quality. The studies were all of high quality, ranging from 7 to 9. The average result was 8.25. The evaluation of the studies' quality is summarized in Figure 2. Sensitivity tests revealed that the aggregate estimations were unaffected significantly by removing any one study at a time.

**TABLE 2:** Bias assessment of included studies.

	Selection					Outcomes		
Author and year of study	Representativeness of sample	Sample size	Non‐respondents/recruitment rate	Ascertainment/exposure	Comparability	Assessment of outcome	Adequacy of follow‐up	Total score
Lehrer et al. 2021 (Lehrer & Rheinstein, 2021)	1	1	1	2	2	1	0	8
Lophatananon et al. 2021 (Lophatananon et al., 2021)	1	1	1	1	1	1	1	7
Wiemken et al. 2021 (Wiemken et al., 2021)	1	1	1	2	2	1	1	9
Lophatananon et al. 2023 (Lophatananon et al., 2023)	1	1	1	2	2	1	1	9

#### 3.3.1 Herpes zoster vaccination and dementia

**FIGURE 3:**
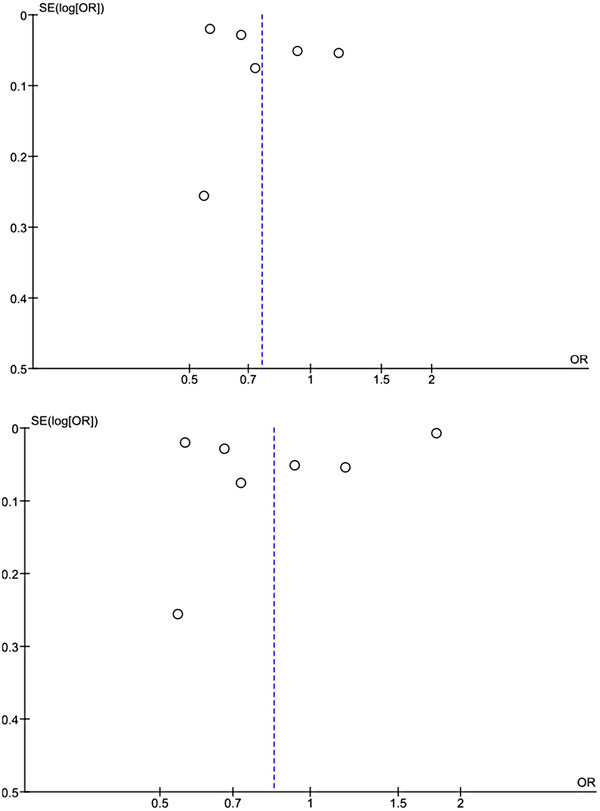
Funnel plot showing publication bias among included studies. OR, odds ratio.

Figure 3 demonstrates the combined effect estimate (HR) for the effect of herpes zoster vaccination on the incidence of dementia. Since high heterogeneity (*I* ^2^ = 100%, *p* < .00001) was observed, the random effects model was used. The pooled OR of 4 studies was 0.91 (95% CI: 0.47, 1.75), *p* (overall effect) = .78), suggesting that herpes zoster vaccination reduces the risk of dementia (Figure 2).

## 4 DISCUSSION

In this meta‐analysis, the association between herpes zoster vaccination and dementia was investigated. Our data demonstrated that patients who had herpes zoster vaccination were at a significantly lower risk of developing dementia. Although a significant heterogeneity (*I* ^2^ = 98%, *p* < .00001) was observed in the meta‐analysis, a pooled OR was estimated at 0.84 (95% CI: 0.50, 1.43), *p* (overall effect) = .53), which indicated that herpes zoster vaccination reduces the risk of dementia. Our study was consistent with the finding in the review and meta‐analysis by Wu et al. (2022) where all vaccinations including herpes zoster were found to be significantly associated with a reduced risk of dementia. In this meta‐analysis, we included two more studies (one cross‐sectional and one cohort study) that were not included in the study by Wu et al. and analyzed the data to show evidence of the association of herpes zoster vaccination with the risk of dementia.

The exact mechanism for the reduced risk of dementia after herpes zoster vaccination is still controversial. HZ immunization may have unique neuroprotective benefits that lessen central nervous system (CNS) inflammation and/or prevent viruses from infecting the brain compared to no HZ vaccination. A latent chronic viral infection is reactivated in zoster illness. It is feasible that preventing clinically confirmed and/or asymptomatic zoster reactivation occurrences through vaccination could lower the associated CNS inflammation and, in turn, lessen the ensuing neuronal damage. Another possible explanation can be that HZ vaccination may decrease abnormal neuroinflammation by reducing proinflammatory cytokines which contribute to the deposition of amyloid‐beta (X. X. Wang et al., 2020; W. Y. Wang et al., 2015). The other possible mechanism could be by reducing the infection. Studies have shown that infectious events may impair cognition and increase the risk of dementia (Douros et al., 2021; Muzambi et al., 2020). This postulation can be supported by the studies done for other vaccinations such as influenza vaccination in the study by Wiemken et al. (2021) and diphtheria, pertussis, and pertussis vaccination in the study by Scherrer et al. (2021) which have shown the role of vaccination on reducing the risk of dementia.

As suggested by the meta‐analysis of Xinhui et al., all vaccinations could reduce the risk of dementia. The capacity of vaccination to lower the risk of contracting infectious diseases is one argument that could apply. Research has indicated that viral episodes can worsen cognitive function and raise the possibility of dementia (Douros et al., 2021; Muzambi et al., 2020). A large multi‐cohort study indicates that infection affecting the central nervous system can raise the risk of dementiaand also reveals that the brain may be impacted by the systemic effects of general inflammation (Sipilä et al., 2021). Recent studies indicate that brain structural alterations and cognitive impairment may still be linked to viral disorders such as COVID‐19, which is mostly characterized by respiratory symptoms (Douaud et al., 2022; Hampshire et al., 2021; Hosp et al., 2021). The relationship between vaccinations and dementia risk, however, may not be entirely explained by eliminating the pertinent pathogens, since vaccines are not equally effective against all pathogens (Wu et al., 2022). Vaccinations may make it possible for the immune system to develop and sustain proper immunological responses, strengthening the body's defense against bacteria and viruses.

The non‐specific effects (NSEs), commonly known as off‐target effects, represent an additional plausible idea (Benn et al., 2013). Numerous basic immunizations were demonstrated to lower childhood all‐cause mortality in studies, supporting the NSEs for vaccinations (McGovern & Canning, 2015). The NSEs hypothesis is further supported by the dose‐dependent connection. Additionally, certain vaccinations may influence the onset of dementia in other, distinct ways. For instance, the herpes zoster vaccine stops the herpes virus from reactivating in the brain; the influenza vaccine lowers cerebrovascular events, a known risk factor for dementia (Raz et al., 2016; Tsivgoulis et al., 2018); and intravenous BCG instillation dramatically raises serum IL‐2 levels, which increases the quantity of neuroprotective Treg cells (Taniguchi et al., 1999). Therefore, it is possible that the vaccines have preventive benefits by interfering in the pathological process of dementia.

This systematic review and meta‐analysis has its own limitations. Since all the studies are retrospective in nature, selection bias could have occurred. The number of studies showing the association between herpes zoster vaccination and dementia risk is less. The reliability of our findings may have been impacted by the short amount of experimental data (gathered from all research) and methodological heterogeneity of the included studies. The results may have been skewed by the varying diagnosis standards for dementia. Regarding the conclusions of the included research's generalizability, caution should also be exercised. There may be regional bias because these investigations were carried out in the United States and the United Kingdom.

## 5 CONCLUSION

Our meta‐analysis has demonstrated that herpes zoster vaccination is significantly associated with a reduction of the risk of dementia. However, more epidemiological studies and experiments are needed to understand the association between herpes zoster vaccination and dementia.

## AUTHOR CONTRIBUTIONS

**Sangam Shah**: Supervision; writing—review and editing conceptualization; methodology; writing—review and editing; writing—original draft. **Krishna Dahal**: Methodology; writing—original draft; writing—review and editing. **Sangharsha Thapa**: Writing—original draft; writing—review and editing; methodology; conceptualization. **Prativa Subedi**: Methodology; formal analysis; writing—review and editing. **Basanta Sharma Paudel**: Methodology; formal analysis; writing—review and editing. **Swati Chand**: Methodology; formal analysis; writing—review and editing. **Amr Salem**: Writing—review and editing; data curation. **Markus Lammle**: Writing—review and editing; supervision. **Ranjit Sah**: Supervision; writing—review and editing. **Martin Krsak**: Supervision; writing—review and editing.

## CONFLICT OF INTEREST STATEMENT

The authors declare no conflicts of interest.

## Funding information

No funding was received for the study.

### PEER REVIEW

The peer review history for this article is available at https://publons.com/publon/10.1002/brb3.3415.

## Data Availability

All the required information is available in the manuscript itself.
