# Correction to Herpes zoster vaccination and the risk of dementia: A systematic review and meta‐analysis

**DOI:** 10.1002/brb3.3474

**Published:** 2024-04-14

Shah, S., Dahal, K., Thapa, S., Subedi, P., Paudel, B. S., Chand, S., Salem, A., Lammle, M., Sah, R., & Krsak, M. (2024). Herpes zoster vaccination and the risk of dementia: A systematic review and meta‐analysis. *Brain and Behavior, 14*, e3415. https://doi.org/10.1002/brb3.3415

The following corrections should be noted for the above article to correct the result in disparity in text and figure of forest and funnel plot.

1. In abstract results, “We evaluated a total of five studies (one cross‐sectional, one case–control, and four cohort studies) that included a total number of 103,615 patients who were vaccinated with herpes zoster vaccine. All the studies were of high quality, ranging from 7 to 9. Due to the high heterogeneity (*I* ^2^ = 100%, *p* < .00001) observed in our study, a random effect model was used for the analysis. The pooled odds ratio was 0.84 (95% CI: 0.50, 1.43), *p* (overall effect) = .53), indicating that herpes zoster vaccination reduces the risk of dementia.” Should read as “We evaluated a total of four studies (one cross‐sectional, one case‐control, and two cohort studies) which included a total number of 941,991 patients who were vaccinated with herpes zoster vaccine. All the studies were of high quality, ranging from 7 to 9. Due to high heterogeneity (*I* ^2^ = 98 %, *p* < .00001) observed in our study, random effect model was used for the analysis. The pooled odds ratio was 0.76 (95% CI: 0.60,0.96), indicating that herpes zoster vaccination reduces the risk of dementia.”
2. In section 3.1, “Following the elimination of duplicate articles, the remaining articles were subjected to title and abstract screening, full‐text screening, and finally, inclusion of five studies that met the inclusion criteria for both a qualitative and quantitative synthesis.” Should read as “Following the elimination of duplicate articles, the remaining articles were subjected to title and abstract screening, full‐text screening, and finally, inclusion of four studies that met the inclusion criteria for both a qualitative and quantitative synthesis.”
3. In section 3.3.1, “Since high heterogeneity (I ^2^ = 100%, p < .00001) was observed, the random effects model was used. The pooled OR of 4 studies was 0.91 (95% CI: 0.47, 1.75), p (overall effect) = .78), suggesting that herpes zoster vaccination reduces the risk of dementia” should read as “Since high heterogeneity (*I* ^2^ = 98%, *p* < .00001) was observed, the random effects model was used. The pooled OR of 4 studies was 0.76 (95% CI: 0.60, 0.96), *p* (overall effect) = 0.02], suggesting that herpes zoster vaccination reduces the risk of dementia.”
4. In section 4, “Although a significant heterogeneity (I ^2^ = 98%, p < .00001) was observed in the meta‐analysis, a pooled OR was estimated at 0.84 (95% CI: 0.50, 1.43), p (overall effect) = .53), which indicated that herpes zoster vaccination reduces the risk of dementia.” Should read as “Although a significant heterogeneity (*I* ^2^ = 98%, *p* < .00001) was observed in the meta‐analysis, a pooled OR was estimated at 0.76 (95% CI: 0.60,0.96), *p* (overall effect) = 0.02], which indicated that herpes zoster vaccination reduces the risk of dementia.”
5. Figure 3 erroneously contained two images. The correct figure appears below.

**FIGURE 3:**
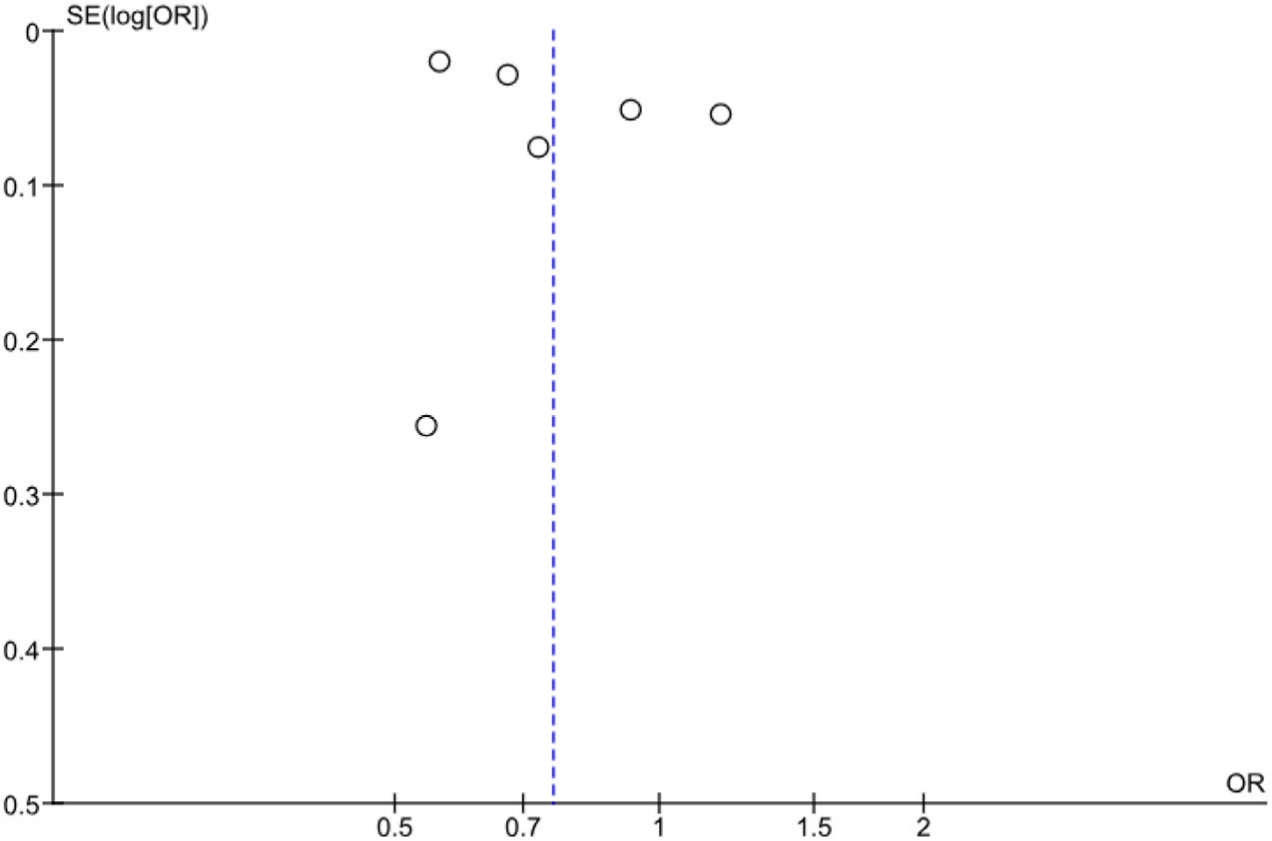
Funnel plot showing publication bias among included studies.

We apologize for this error.

